# Identification and verification of ferroptosis-related genes in the pathology of epilepsy: insights from CIBERSORT algorithm analysis

**DOI:** 10.3389/fneur.2023.1275606

**Published:** 2023-10-31

**Authors:** Dan Xu, ManMan Chu, YaoYao Chen, Yang Fang, JingGuang Wang, XiaoLi Zhang, FaLin Xu

**Affiliations:** ^1^Department of Pediatric Neurology, The Third Affiliated Hospital of Zhengzhou University, Zhengzhou, China; ^2^Department of Laboratory Medicine, The Third Affiliated Hospital of Zhengzhou University, Zhengzhou, China; ^3^Department of Pediatrics, Third Affiliated Hospital of Zhengzhou University, Zhengzhou, China

**Keywords:** ferroptosis, epilepsy, bioinformatic analysis, differentially expressed genes, immune landscape

## Abstract

**Background:**

Epilepsy is a neurological disorder characterized by recurrent seizures. A mechanism of cell death regulation, known as ferroptosis, which involves iron-dependent lipid peroxidation, has been implicated in various diseases, including epilepsy.

**Objective:**

This study aimed to provide a comprehensive understanding of the relationship between ferroptosis and epilepsy through bioinformatics analysis. By identifying key genes, pathways, and potential therapeutic targets, we aimed to shed light on the underlying mechanisms involved in the pathogenesis of epilepsy.

**Materials and methods:**

We conducted a comprehensive analysis by screening gene expression data from the Gene Expression Omnibus (GEO) database and identified the differentially expressed genes (DEGs) related to ferroptosis. Gene Ontology (GO) and Kyoto Encyclopedia of Genes and Genomes (KEGG) analyses were performed to gain insights into the biological processes and pathways involved. Moreover, we constructed a protein–protein interaction (PPI) network to identify hub genes, which was further validated using the receiver operating characteristic (ROC) curve analysis. To explore the relationship between immune infiltration and genes, we employed the CIBERSORT algorithm. Furthermore, we visualized four distinct interaction networks—mRNA–miRNA, mRNA–transcription factor, mRNA–drug, and mRNA–compound—to investigate potential regulatory mechanisms.

**Results:**

In this study, we identified a total of 33 differentially expressed genes (FDEGs) associated with epilepsy and presented them using a Venn diagram. Enrichment analysis revealed significant enrichment in the pathways related to reactive oxygen species, secondary lysosomes, and ubiquitin protein ligase binding. Furthermore, GSVA enrichment analysis highlighted significant differences between epilepsy and control groups in terms of the generation of precursor metabolites and energy, chaperone complex, and antioxidant activity in Gene Ontology (GO) analysis. Furthermore, during the Kyoto Encyclopedia of Genes and Genomes (KEGG) pathway analysis, we observed differential expression in pathways associated with amyotrophic lateral sclerosis (ALS) and acute myeloid leukemia (AML) between the two groups. To identify hub genes, we constructed a protein–protein interaction (PPI) network using 30 FDEGs and utilized algorithms. This analysis led to the identification of three hub genes, namely, HIF1A, TLR4, and CASP8. The application of the CIBERSORT algorithm allowed us to explore the immune infiltration patterns between epilepsy and control groups. We found that CD4-naïve T cells, gamma delta T cells, M1 macrophages, and neutrophils exhibited higher expression in the control group than in the epilepsy group.

**Conclusion:**

This study identified three FDEGs and analyzed the immune cells in epilepsy. These findings pave the way for future research and the development of innovative therapeutic strategies for epilepsy.

## 1. Introduction

Epilepsy is a chronic neurological disorder characterized by recurrent and unpredictable seizures. Seizures are caused by abnormal electrical activity in the brain, which can result in sudden and uncontrolled bursts of abnormal brain cell firing, leading to various symptoms (1, 2). These symptoms can be mild, such as brief periods of impaired consciousness, confusion, or altered sensations. They can also be severe, including muscle convulsions, fainting, loss of consciousness, generalized convulsions, and foaming at the mouth (3). In recent years, our understanding of the pathogenesis of epilepsy has deepened, although the exact mechanisms behind its onset are still unclear (4). To provide more precise guidance for preventing and treating epilepsy, it is necessary to unravel its pathogenesis at the molecular level. In this study, we utilized a dataset of epilepsy from the Gene Expression Omnibus (GEO) database and conducted bioinformatics analysis on the genes associated with epilepsy.

Ferroptosis is a similar form of cell death with distinctive biological features to other common cell death mechanisms. A prominent feature of ferroptosis is the accumulation of free iron within cells resulting in an irreversible lipid peroxidation reaction (5). Excessive accumulation of intracellular ions can lead to lipid oxidation, membrane damage, and functional impairment, ultimately resulting in cell death. Factors involved in the regulation of ferroptosis include lipid peroxidation products, antioxidants, and iron-related proteins, as well as molecules and pathways associated with cell survival and death (6). Significant progress has been made in the study of ferroptosis, particularly in understanding its regulatory mechanisms, related signaling pathways, and its association with the development of epilepsy (7, 8). These studies contribute to elucidating the deeper mechanisms underlying cell death and provide theoretical and practical foundations for developing novel therapeutic approaches for epilepsy.

Gene set variation analysis (GSVA) is a non-parametric unsupervised analysis method primarily used to transform gene expression matrices into gene set expression matrices for the evaluation of transcriptional enrichment of different metabolic pathways among different samples (9, 10). To investigate the biological process variations between epilepsy patients and normal controls, the R package “GSVA” can be employed for gene set variation analysis based on gene expression profiling datasets from different epilepsy patients and normal controls.

Ferroptosis plays an important role in epilepsy, and this research aims to identify key genes associated with epilepsy and ferroptosis, which may serve as novel biomarkers or possible therapeutic targets for epilepsy. In this study, the CIBERSORT algorithm was performed to evaluate the immune infiltration characteristics of epilepsy and normal samples in the integrated dataset (GSE32534 and GSE143272), assessing the composition of 22 immune cell types in each group. The selected hub genes were then associated with the levels of immune cell infiltration using Pearson correlation coefficients and significance levels. Finally, a compound network, drug network, mRNA–miRNA network, and transcription factor network of the three hub genes were constructed. This study provides a research foundation for exploring potential regulatory targets and possible mechanisms of epilepsy, offering new insights into the treatment of this disease.

## 2. Materials and methods

### 2.1. Data source and preprocessing

Ferroptosis genes were selected from the GeneCards database (11). Gene expression data of epilepsy including GSE32534 and GSE143272 were obtained from the Gene Expression Omnibus (GEO) database (12, 13). GSE32534 is based on the GPL570 platform, which includes 10 tissue samples, with five being Epilepsy patients and five being normal controls. The other dataset GSE143272 is based on the GPL10558 platform and includes 34 epilepsy patients and 50 normal controls. The detailed information on both datasets is shown in Table 1.

**Table 1:** Data set information.

ID	GPL	Sample source	Sample size	References	Species
GSE32534	GPL570	FFPE peritumoral sections	10 (5 Epilepsy, 5 Normal)	PMID: 23418513	*Homo sapiens*
GSE143272	GPL10558	Whole blood	85 (34 Epilepsy, 51 Normal)	PMID: 30826443	*Homo sapiens*
				PMID: 32054883	

### 2.2. Differentially expressed genes between group identification

The raw data of microarray datasets underwent background correction and quantile normalization using the Robust Multichip Average (RMA) method, enhancing data quality and minimizing potential variations. Probe IDs were converted to gene names based on the annotation platform. Probes without corresponding gene names or genes with multiple probes were either removed or averaged. The batch effects in the GSE32534 and GSE143272 datasets were accounted for using the sva package. Differentially expressed genes (DEGs) were identified using the limma package with a threshold set at|logFC| > 0.2 and *p* < 0.05. Additionally, principal component analysis (PCA) was used to visualize the gene expression differences between epilepsy and normal groups. Volcano plots and heatmaps of the DEGs were generated using ggplot2 and the complex heatmap package in R.

### 2.3. Gene function enrichment analysis

The interaction between ferroptosis death-related genes and DEGs was identified for further research. Gene Ontology (GO) and Kyoto Encyclopedia of Genes and Genomes (KEGG) were performed using the “clusterProfiler” package of R with a value of *p* < 0.05 and FDR (*q*.value) < 0.25. Gene set enrichment analysis (GSEA) was used to estimate the changes in pathway and biological process activity. To investigate biological process variations between epilepsy patients and normal controls, we employed the “GSVA” R package to analyze gene set variations (GSVA). We downloaded reference gene sets “c2.cp.kegg.v7.5.1.symbols.gmt” and “c5.go.v7.5.1.symbols.gmt” from MSigDB database. A *t*-test was performed to identify significant differences between the epilepsy and normal groups, with a significance threshold of value of *p* < 0.05 (Table 2).

**Table 2:** GO enrichment analysis.

Category	GO	Description	*p* value	Adjust *p* value	*Q* value
GO Biological processes	GO:0071496	Cellular response to external stimulus	1.21E−10	1.98E−07	1.22E−07
GO Biological processes	GO:0006979	Response to oxidative stress	3.02E−09	2.02E−06	1.25E−06
GO Biological processes	GO:0000302	Response to reactive oxygen species	3.70E−09	2.02E−06	1.25E−06
GO Biological processes	GO:0072593	Reactive oxygen species metabolic process	6.61E−09	2.70E−06	1.67E−06
GO Biological processes	GO:2000377	Regulation of reactive oxygen species metabolic process	9.07E−09	2.97E−06	1.83E−06
GO Cellular components	GO:0071682	Endocytic vesicle lumen	7.58E−06	0.001160277	0.000766333
GO Cellular components	GO:0031838	Haptoglobin-hemoglobin complex	0.00015053	0.007677017	0.005070476
GO Cellular components	GO:0044754	Autolysosome	0.00015053	0.007677017	0.005070476
GO Cellular components	GO:0030139	Endocytic vesicle	0.000232601	0.008897005	0.005876247
GO Cellular components	GO:0005767	Secondary lysosome	0.000369867	0.01131792	0.0074752
GO Molecular function	GO:0031625	Ubiquitin protein ligase binding	8.02E−07	9.96E−05	6.26E−05
GO Molecular function	GO:0044389	Ubiquitin-like protein ligase binding	1.21E−06	9.96E−05	6.26E−05
GO Molecular function	GO:0016209	Antioxidant activity	1.65E−05	0.000903281	0.000568174
GO Molecular function	GO:0004601	Peroxidase activity	0.000103744	0.004253512	0.002675508
GO Molecular function	GO:0016684	Oxidoreductase activity, acting on peroxide as acceptor	0.000130057	0.004265876	0.002683285

### 2.4. Identification of gene clusters

The STRING database was applied to construct the PPI network and then was visualized by the Cytoscape software. To visualize molecular interaction networks, we carefully selected critical nodes. Moreover, utilizing the cytoHubba plugin, we identified hub genes based on the protein–protein interaction (PPI) network. To perform differential expression analysis of hub genes, a *t*-test was applied, and the receiver operating characteristic (ROC) curve generated by the pROC package was used to assess the predictive accuracy of hub genes for epilepsy disease.

### 2.5. Analysis of hub gene network

To further enrich the FDEG framework, we constructed four distinct interaction networks: mRNA–miRNA, mRNA–transcription factor, mRNA–drug, and mRNA–compound networks. The mRNA–miRNA interaction network was built using the TargetScan database.^1^ This database provides comprehensive miRNA target predictions for mRNA transcripts. The mRNA transcription factor network was constructed utilizing the ChEA3 online tool. The mRNA–drug network was established based on the DGIdb database,^2^ which contains information on drug–gene interactions. The CTD database^3^ offers information on chemical–gene interactions, which was used to generate the mRNA–compound network. The network relationships in the four types of interactions were visualized using the graph package.

### 2.6. Landscape of immune infiltration of epilepsy

**Figure 1:**
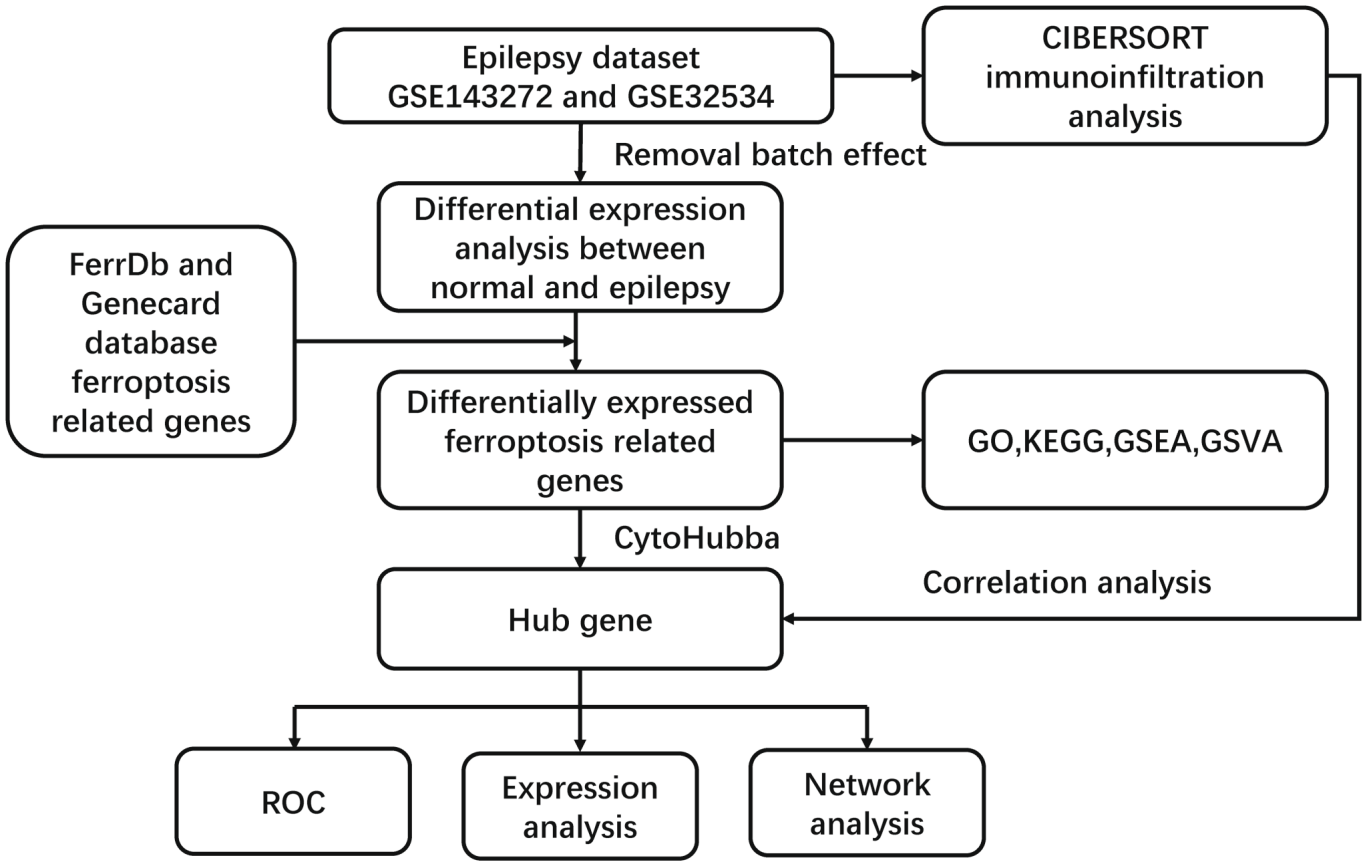
The flowchart of this study.

The CIBERSORT algorithm was used to infer the relative proportions of 22 infiltrating immune cells using standardized gene expression data. Pearson’s correlation analysis was performed between the selected hub genes and the infiltration levels of the 22 immune cells with a significance threshold of *p* < 0.05. The lollipop plots were used to analyze the association between each hub gene and the immune cells using the ggplot2 package (Figure 1).

### 2.7. Statistical analysis

Spearman’s rank tests or Pearson’s correlation coefficient was used to analyze the correlation between genes and immune cells. Multiple testing adjustment was performed using the Benjamini–Hochberg (BH) method to control the false discovery rate (FDR) in multiple tests. Independent Student’s *t*-test was used to estimate the statistical significance of normally distributed variables. Wilcoxon rank-sum test was analyzed to compare continuous variables. R software (version 4.1.1) was applied to perform the statistical analysis. All statistical *p*-values were two-tailed, and values of *p* < 0.05 were considered statistically significant.

## 3. Results

### 3.1. Identification of differentially expressed ferroptosis-related genes

**Figure 2:**
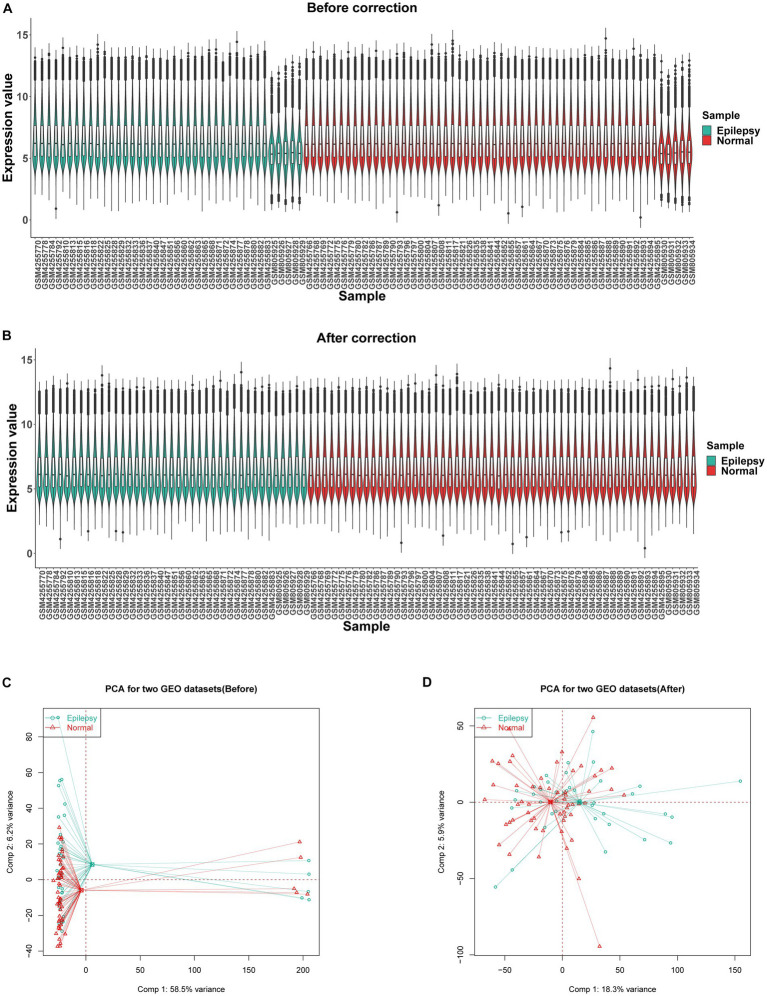
Data preprocessing. Box plots showing the distribution of gene before **(A)** and after **(B)** batch correction. **(C)** Two-dimensional PCA scatter plot of the integrated dataset (GSE32534 and GSE143272) before correction, displaying poor separation between epilepsy and normal samples. **(D)** Two-dimensional PCA scatter plot of the integrated dataset (GSE32534 and GSE143272) after correction, displaying better separation between epilepsy and normal samples.

**Figure 3:**
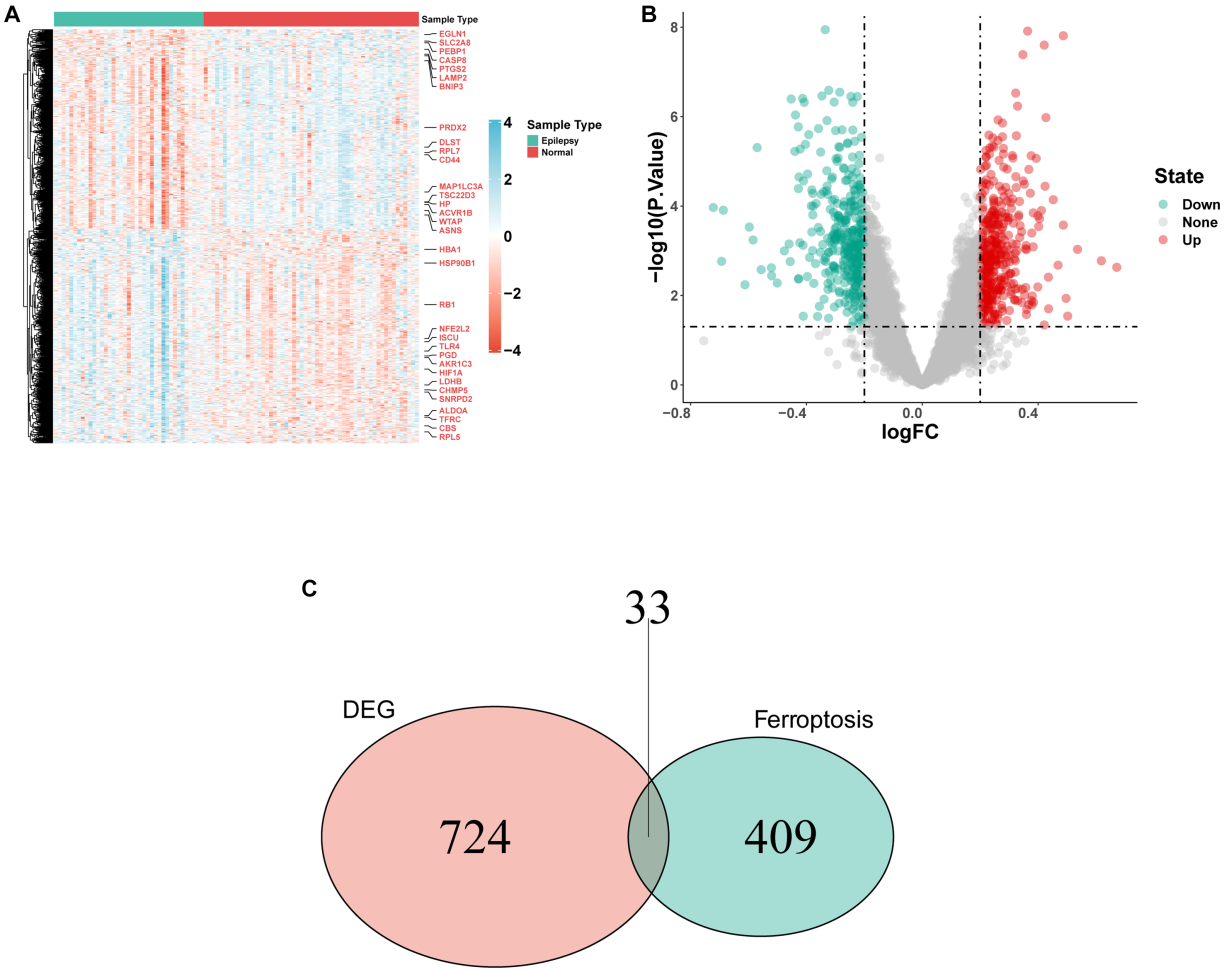
FRDEGs in the integrated dataset. **(A)** The heatmap of FRDEGs between epilepsy and normal samples, including 392 upregulated genes and 365 downregulated genes. **(B)** Volcano plot depicting the differential expression analysis between epilepsy and normal samples. **(C)** After the intersection, 33 FRDEGs were identified based on the Venn diagram.

GSE32534 and GSE143272 were downloaded from the GEO database. The specific information regarding these two datasets is provided in Table 1. First, the “sva” package helped in removing batch effects of the datasets. Figures 2A,B show that the heterogeneity of the two databases was eliminated. The two-dimensional PCA scatter plots (Figures 2C,D) also demonstrate significant differences between epilepsy and normal groups after batch effect correction. In addition, the difference between the two groups was compared using GEO2R, and the results were downloaded for further research. Totally, 757 DEGs were detected, of which 365 were downregulated and 392 were upregulated genes (Figure 3A). Then, the ferroptosis-related genes were extracted from the “FerrDb” database. A volcano plot was performed to validate the results (Figure 3B). A total of 33 FDEGs were selected based on the Venn diagram (Figure 3C).

### 3.2. Enrichment analysis of ferroptosis-related DEGs and PPI network analysis

**Figure 4:**
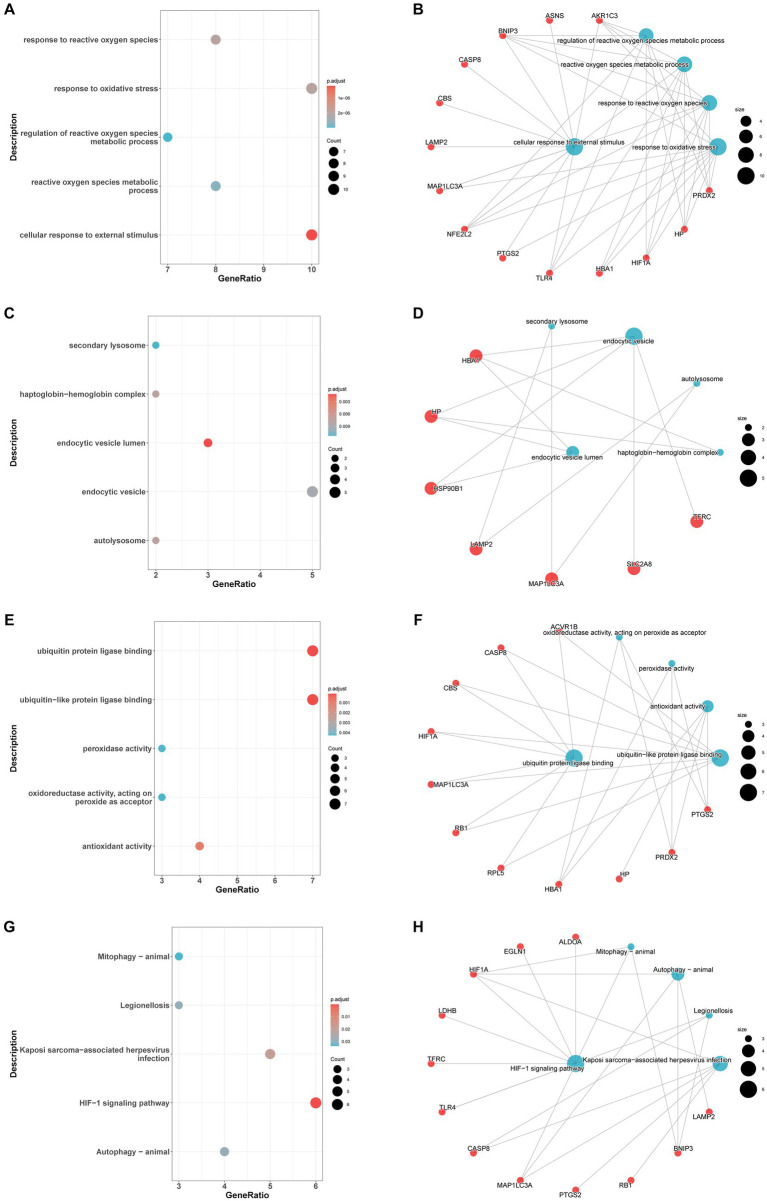
Enrichment analysis of FRDEGs. GO-biological process enrichment analysis of the 33 FDEGs with bubble plot **(A)** and network plot **(B)**. GO-cellular component enrichment analysis of the 33 FDEGs with bubble plot **(C)** and network plot **(D)**. GO-molecular function enrichment analysis of the 33 FDEGs with bubble plot **(E)** and network plot **(F)**. KEGG pathways enrichment analysis of the 33 FDEGs with bubble plot **(G)** and network plot **(H)**.

**Figure 5:**
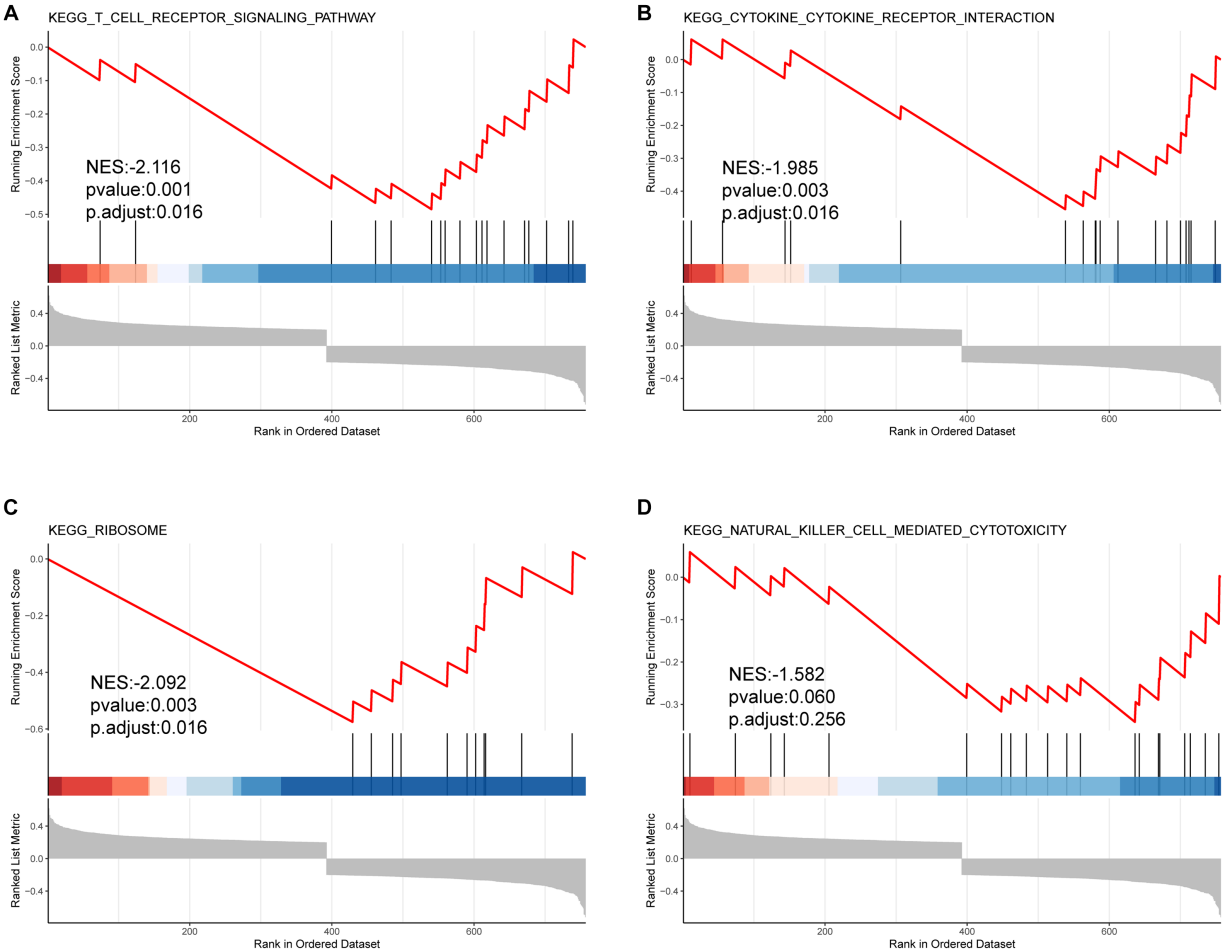
GSEA results of FRDEGs. The top four enriched pathways including **(A)** KEGG T cell receptor signaling pathway **(A)**, KEGG cytokine receptor interaction **(B)**, KEGG ribosome **(C)**, and KEGG natural killer cell mediated cytotoxicity **(D)**.

In the GO-BP analysis (Figures 4A,B), the main pathways were response to reactive oxygen species, response to oxidative stress, and regulation of reactive oxygen species metabolic process. In the GO-CC analysis (Figures 4C,D), the significantly enriched terms were secondary lysosome, endocytic vesicle lumen, and endocytic vesicle. In the GO-MF analysis (Figures 4E,F), these genes are mainly enriched in ubiquitin protein ligase binding, ubiquitin-like protein ligase blinding, and peroxidase activity. In KEGG analysis (Figures 4G,H), 33 FDEGs were mostly associated with legionellosis, HIF-1 signaling pathway, and autophagy-animal. Moreover, the GSEA (Figures 5A–D) was applied to the top four pathways, namely, T-cell receptor signaling pathway, cytokine receptor interaction, ribosome, and natural killer cell-mediated cytotoxicity (Table 3).

**Table 3:** KEGG enrichment analysis.

Category	KEGG	Description	*p* value	Adjust *p* value	*Q* value
KEGG	hsa04066	HIF-1 signaling pathway	9.17E−07	0.000111	7.72E−05
KEGG	hsa05167	Kaposi sarcoma-associated herpesvirus infection	0.000316	0.019125	0.01331
KEGG	hsa05134	Legionellosis	0.00075	0.028758	0.020014
KEGG	hsa04140	Autophagy—animal	0.000951	0.028758	0.020014
KEGG	hsa04137	Mitophagy—animal	0.001481	0.033603	0.023386

### 3.3. Gene set variation analysis

**Figure 6:**
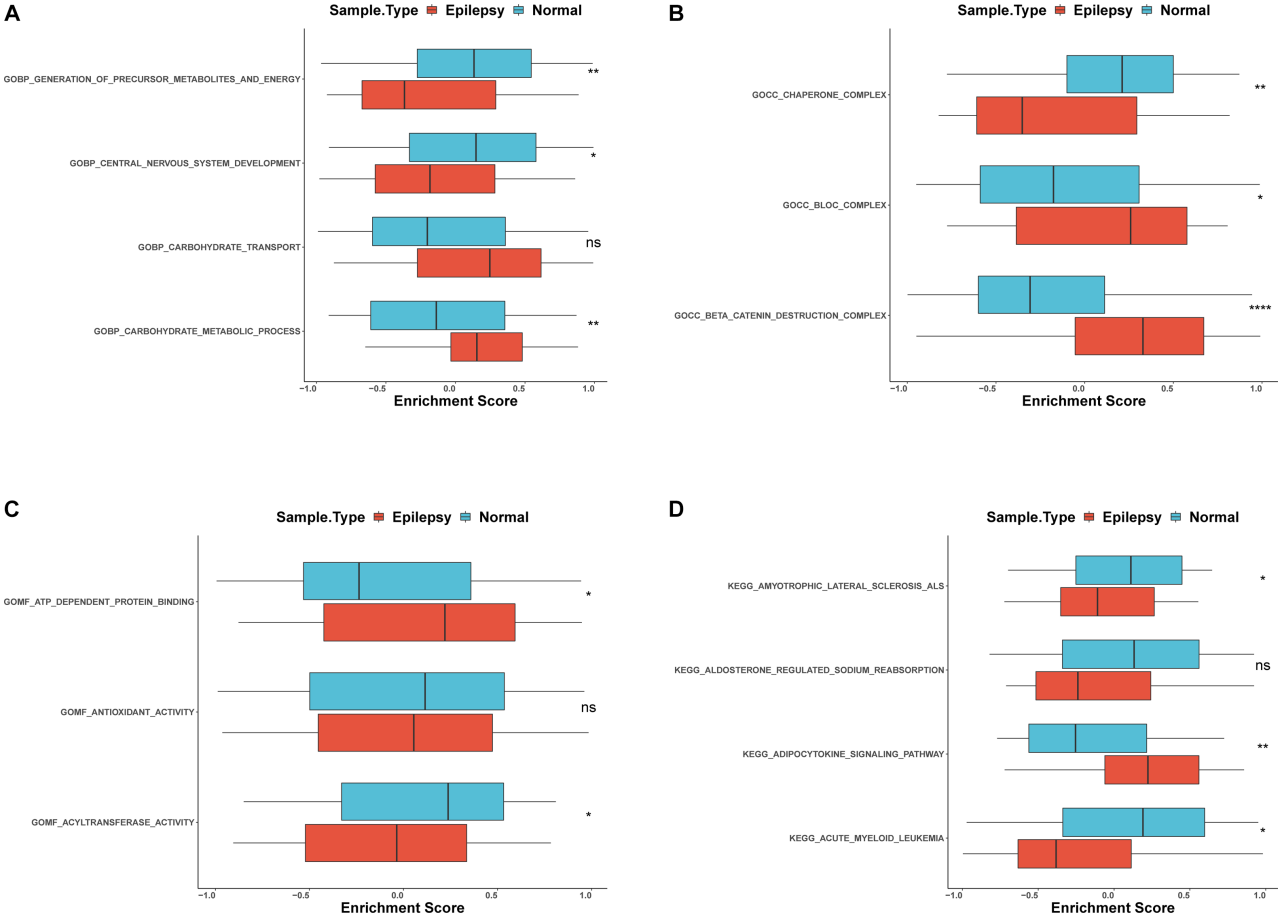
GSVA analysis of FRDEGs **(A)** Differential enrichment of GO biological process between epilepsy and normal groups with box plot **(B)**. Differential enrichment of GO cellular components between epilepsy and normal groups with box plot **(C)**. Differential enrichment of GO molecular function between epilepsy and normal groups with box plot **(D)**. KEGG pathways between epilepsy and normal groups with box plot. ns: non-significant,*: *p* < 0.05, **: *p* < 0.01, ***: *p* < 0.001, ****: *p* < 0.00001.

To further identify the function of these genes, the GSVA enrichment analysis was carried out and revealed that the generation of precursor metabolites and energy, chaperone complex, and antioxidant activity (Figures 6A–C) were significant differences between epilepsy and control groups in GO analysis. In the KEGG pathway, amyotrophic lateral sclerosis (ALS) and acute myeloid leukemia were different expressions between the two groups (Figure 6D). These biological functions and pathways play a crucial role in the occurrence and development of epilepsy (Table 4).

**Table 4:** Gene set enrichment analysis results.

Description	Enrichment score	*p* value	Adjust *p* value	*Q* value
KEGG_T_CELL_RECEPTOR_SIGNALING_PATHWAY	−0.484842528	0.00104261	0.015781717	0.013680746
KEGG_CYTOKINE_CYTOKINE_RECEPTOR_INTERACTION	−0.454764294	0.002545783	0.015781717	0.013680746
KEGG_RIBOSOME	−0.575067024	0.002785009	0.015781717	0.013680746
KEGG_NATURAL_KILLER_CELL_MEDIATED_CYTOTOXICITY	−0.34135783	0.060329068	0.256398537	0.222264986

### 3.4. Identification of gene clusters

**Figure 7:**
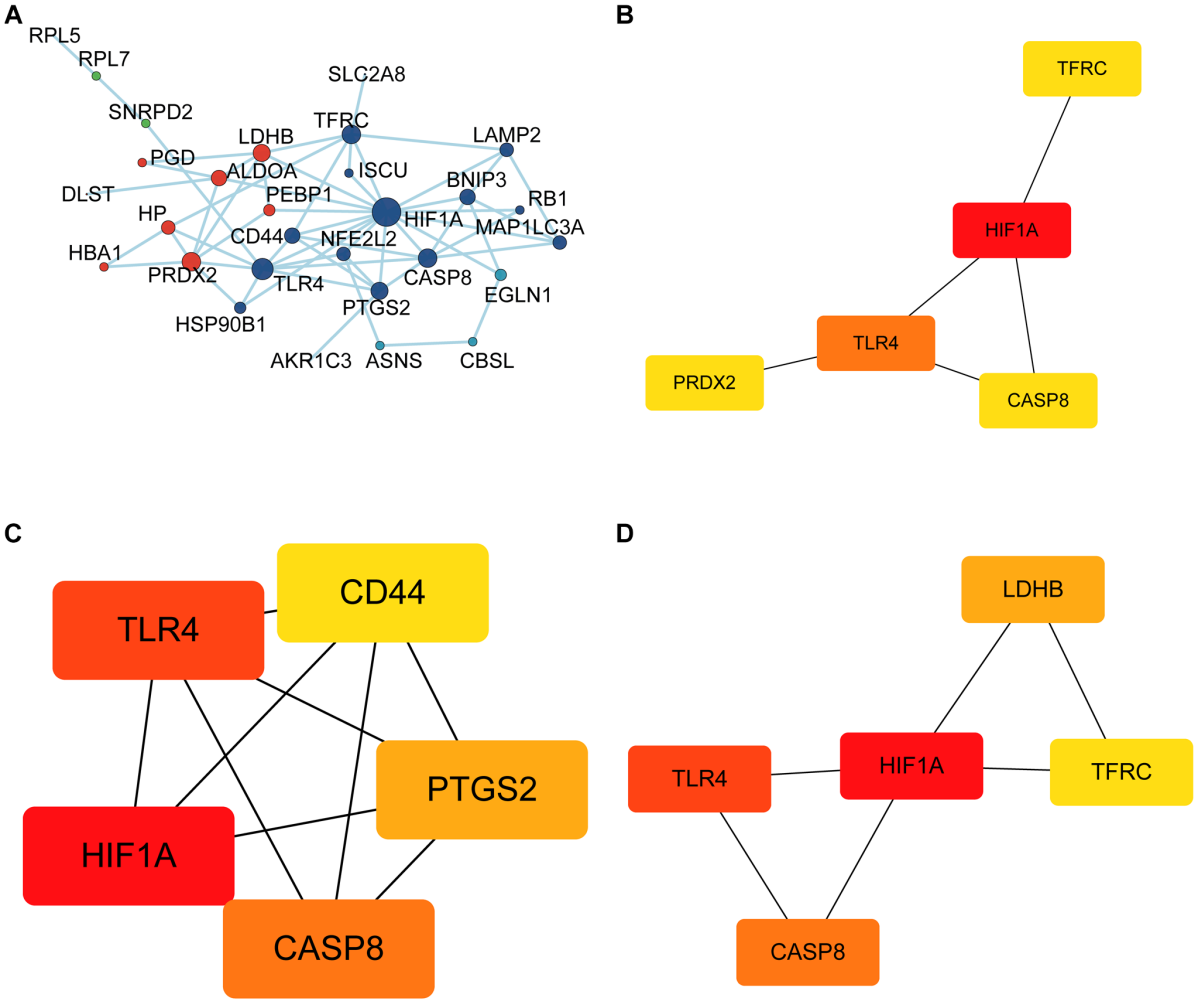
Identification of hub genes. **(A)** Protein-protein interaction network of FRDEGs. The top 5 hub genes were selected using the Degree algorithm **(B)**, MCC algorithm **(C)** and MNC algorithm **(D)**.

**Figure 8:**
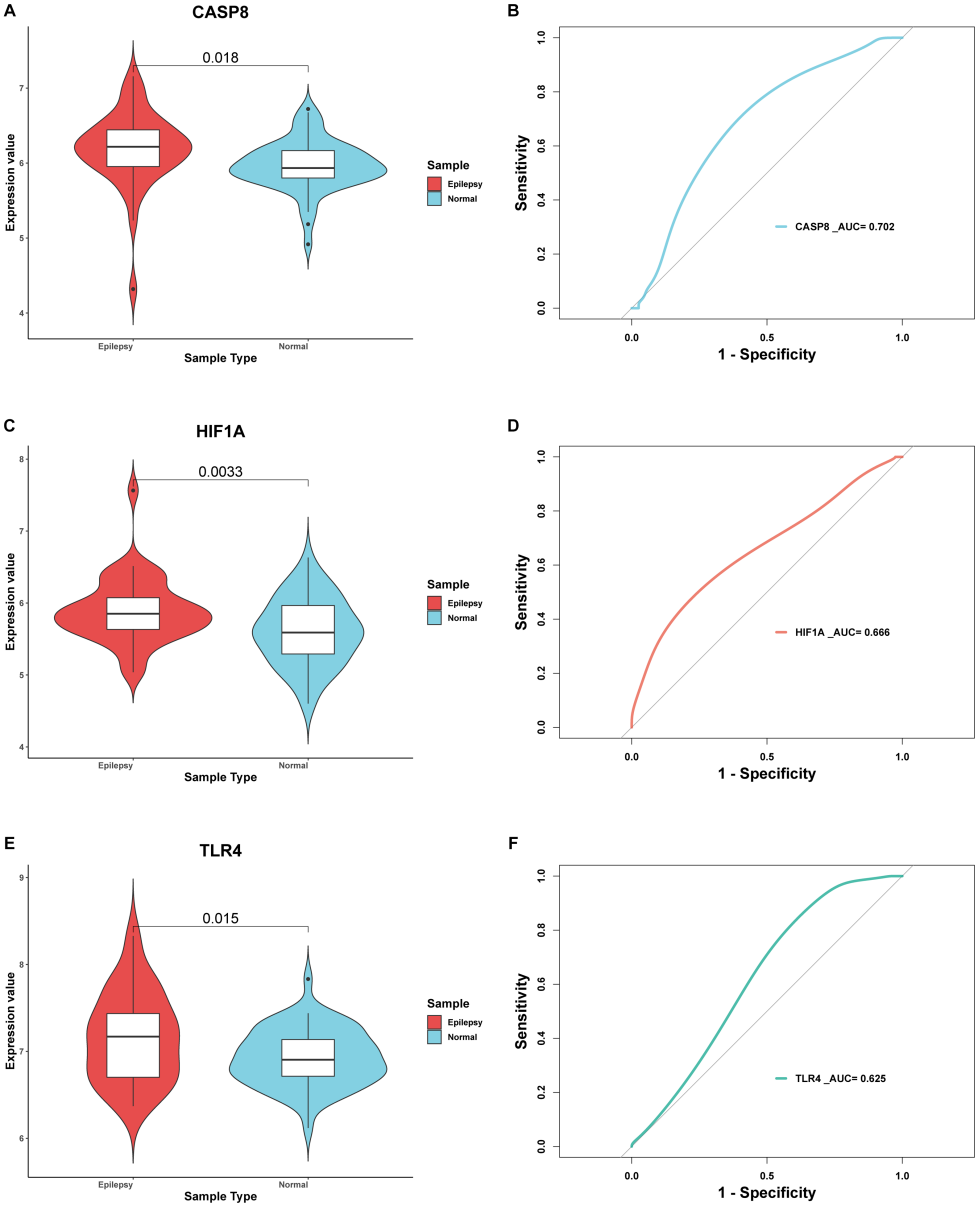
Evaluation of 3 hub genes. **(A)** *t*-test and ROC curve analysis **(B)** of CASP8 gene in distinguishing epilepsy. **(C)** *t*-test and ROC curve analysis **(D)** of HIF1A gene in distinguishing epilepsy **(E)** *t*-test and ROC curve analysis **(F)** of TLR4 gene in distinguishing epilepsy. *p*-value < 0.05 was considered statistically significant.

In total, 30 of 33 FDEGs were used to apply the PPI network (Figure 7A). Through the degree, MCC, and MNC algorithms, we detected hub genes for further analysis. According to the degree algorithm (Figure 7B), the top five genes were TFRC, HIF1A, TLR4, CASP8, and PRDX2. Using the MCC algorithm (Figure 7C), the top five genes were CD44, HIF1A, TLR4, CASP8, and PTGS2. However, the top five hub genes screened by the MNC algorithm were LDHB, HIF1A, TLR4, CASP8, and TFRC (Figure 7D). Finally, the intersection of hub genes screened by three algorithms was obtained, namely, HIF1A, TLR4, and CASP8. To verify the predictive significance of the three genes, *t*-tests and ROC curves were performed on the two datasets; the *t*-test shows the CASP8, HIF1A, and TLR4 genes were significantly differentially expressed between epilepsy and control groups in the integrated dataset with *p* < 0.05 (Figures 8A,C,E). Meanwhile, the AUC values of the three hub genes were all greater than 0.6 (Figures 8B,D,F), indicating that the three hub genes can still effectively distinguish epilepsy from the control group.

### 3.5. Immune cell infiltration analysis

**Figure 9:**
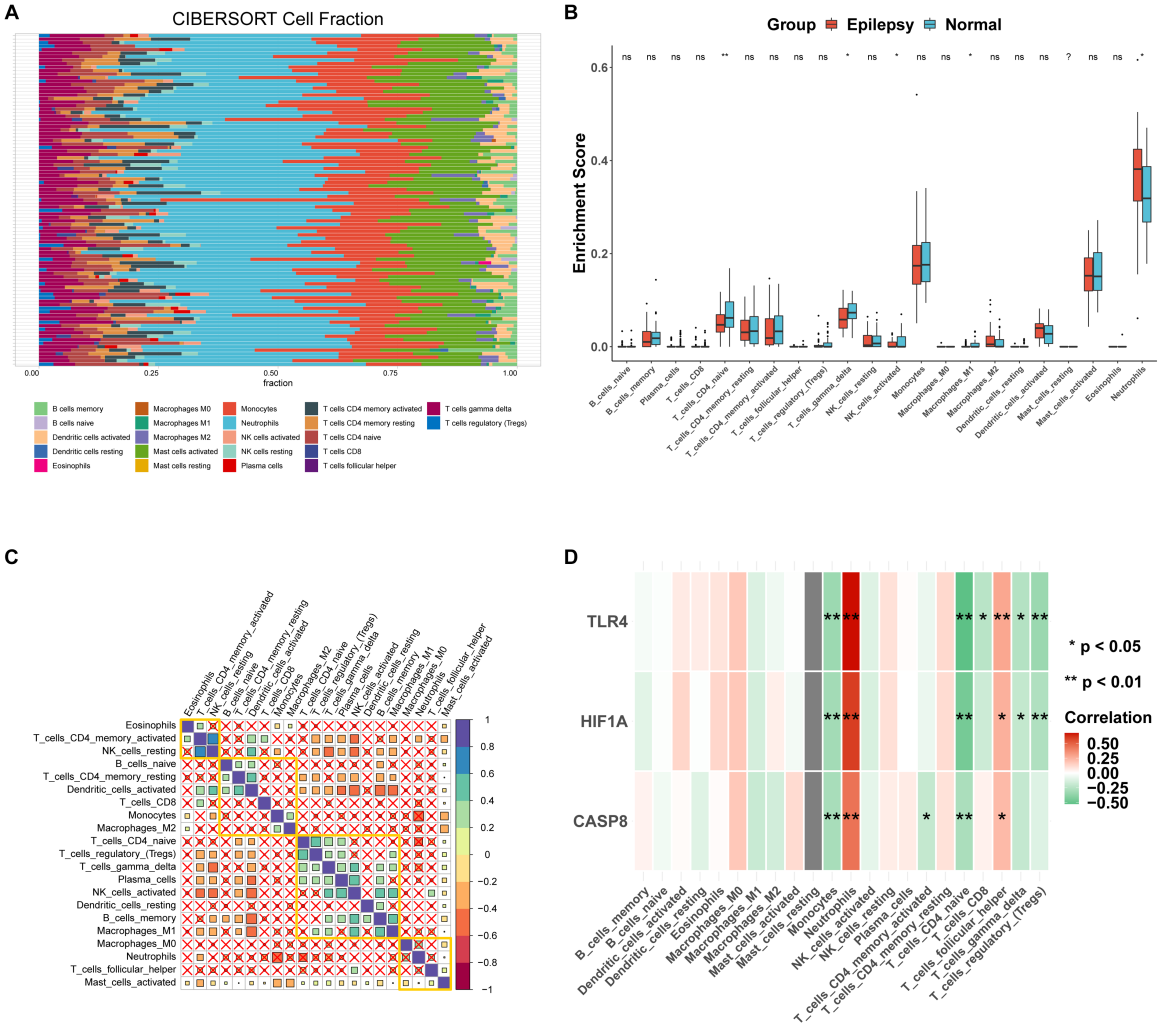
Immune infiltration analysis of the integrated dataset. **(A)** Stacked bar plot showing the proportion of 21 kinds of immune cell in the integrated dataset. **(B)** Box plot analysis of the differences in immune cell abundance between epilepsy and normal samples. **(C)** Correlation analysis of 21 immune cell types in the integrated dataset, with an X indicated *p* > 0.05, blue indicated positive correlation, and red indicated negative correlation. **(D)** The correlation between hub genes and 21 immune cell types with lollipop plot. ns: non-significant, *: *p* < 0.05, **: *p* < 0.01, ***: *p* < 0.001, ****: *p* < 0.00001.

**Figure 10:**
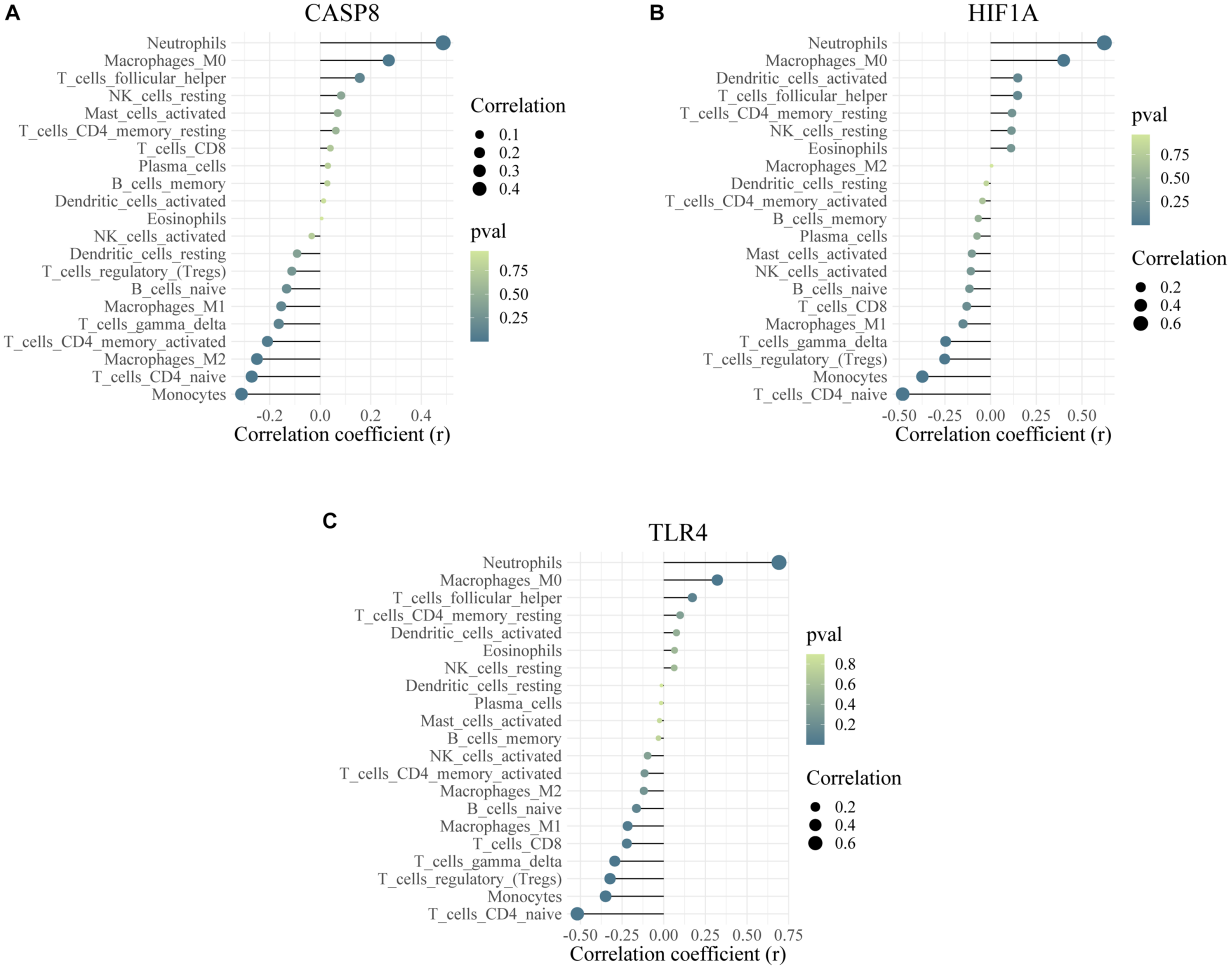
Correlation analysis between hub genes and immune cells. The association of CASP8 gene **(A)**, HIF1A gene **(B)** and TLR4 gene **(C)** with 21 kinds of immune cell.

CIBERSORT was performed to identify the types of immune cells involved in the formation of epilepsy. In total, 22 kinds of immune cell types were obtained for analysis, and immune cells in different groups were significantly different (Figure 9A). The proportion of CD4-naïve T cells, gamma delta T cells, M1 macrophages, and neutrophils was higher in the control group than the epilepsy group (Figure 9B). Correlation analysis revealed a significant difference (*p* < 0.05) between mast cells activated cells and 20 other immune cells (Figure 9C). Figure 9D shows the correlation between three hub genes and immune cells. CASP8, HIF1A, and TLR4 were significantly correlated with monocytes, neutrophils, CD4-naïve T cells, and follicular helper T cells. In addition, the correlation between CASP8 (Figure 10A), HIF1A (Figure 10B), TLR4 (Figure 10C) genes, and 21 immune cells be directly perceived through the lollipop maps.

### 3.6. Network analysis of hub genes

**Figure 11:**
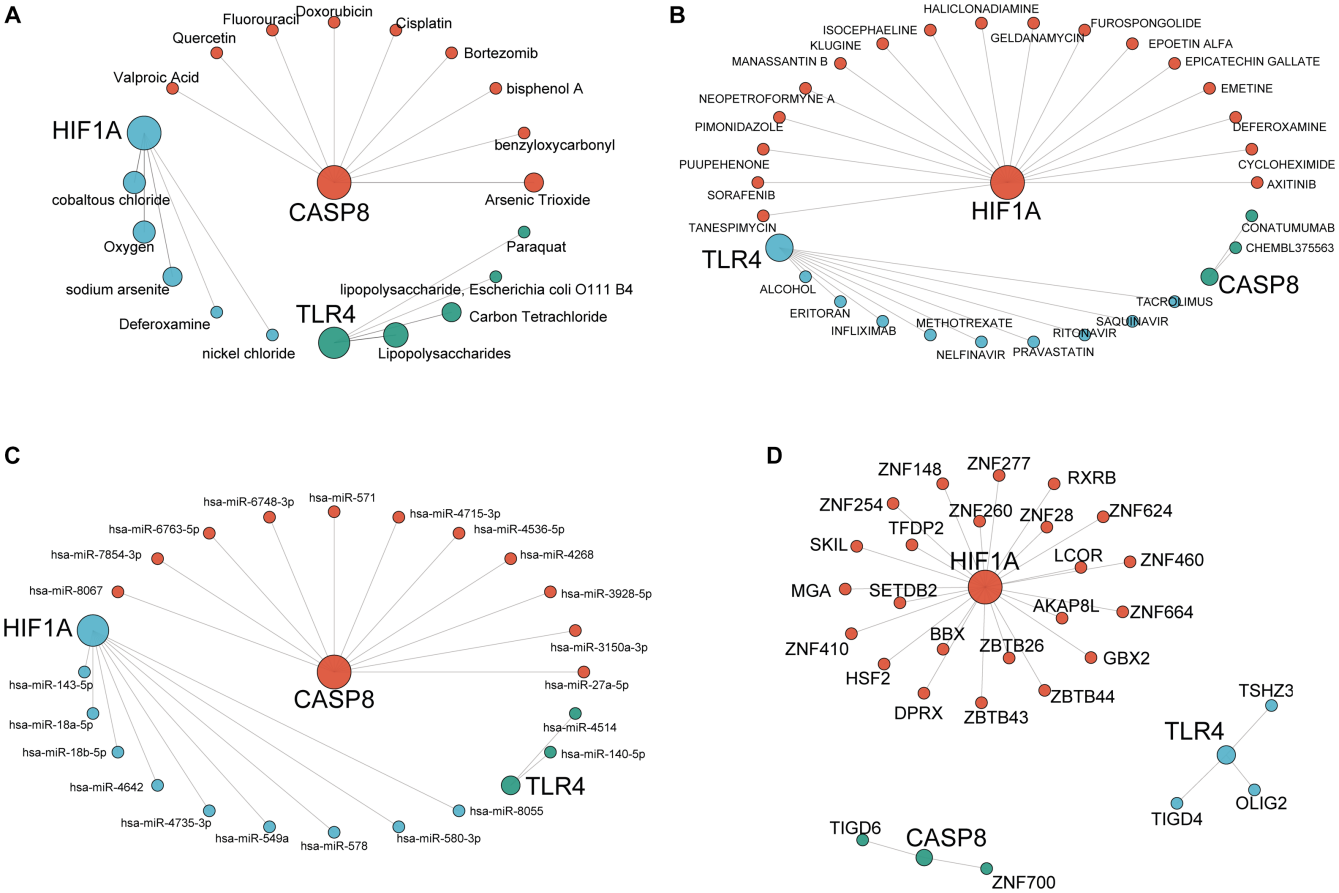
Construction of the interaction network for hub genes. mRNA-compound network **(A)**, mRNA-drug network **(B)**, mRNA-miRNA network **(C)**, and mRNA-transcription factor network **(D)**. The largest nodes in the network represent the hub genes, and each color represents the interaction relationship of each gene.

The database CTD was adopted for predicting the target compound of the key genes. The possible mRNA–compound network was constructed to explore the environmental exposure factors related to the three ferroptosis-driver FDEGs (Figure 11A). We also performed the mRNA–drug networks to predict the potential mechanisms of drug action (Figure 11B). Figure 11C shows the mRNA–miRNA network investigated the molecular mechanism in the FDEGs. It was hypothesized that transcription factors in the regulatory network might play a significant role in epilepsy, so the mRNA–transcription factor networks were constructed (Figure 11D).

## 4. Discussion

Epilepsy is one of the most common chronic neurological disorders. Moreover, it is a complex disease primarily caused by genetic and environmental factors, such as a family history of epilepsy, neurological comorbidities, premature birth, maternal alcohol abuse, and smoking during pregnancy. The identification of candidate biomarkers and the understanding of the pathogenic mechanisms are crucial for early diagnosis and effective treatment of epilepsy (14). Moreover, ferroptosis is associated with various diseases, such as tumors and neurological disorders. Ferroptosis is a form of cell death that can cause damage to cells and tissues when there is an excessive amount of iron ions (15). In epilepsy, excessive ferroptosis may be involved in the occurrence and progression of the disease (16). However, the specific mechanisms and role of ferroptosis in these diseases and their treatments require further research and exploration.

In our research, we identified the differentially expressed genes related to ferroptosis in epilepsy patients. A total of 33 differentially expressed genes were identified in epilepsy, and these findings suggest the involvement of ferroptosis-related genes in the pathogenesis of epilepsy and indicate potential pharmacological targets (17, 18). To understand the role of these ferroptosis-related genes in epilepsy, GO enrichment analyses were conducted. The results revealed that the biological process is primarily involved in pathways associated with response to reactive oxygen species or oxidative stress and regulation of reactive oxygen species metabolic process. The cellular component analysis predominantly involves the secondary lysosome, endocytic vesicle lumen, and endocytic vesicle. In terms of molecular function, these genes are mainly enriched in ubiquitin protein ligase binding, ubiquitin-like protein ligase blinding, and peroxidase activity, which is consistent with previous studies (19). In previous studies, some antioxidant drugs have already been used in medical use, which provided potential treatment for reducing the seizure burden (20). A study indicated that (21) abnormal synaptic transmission regulation is crucial in the development of various brain disorders, including epilepsy. In epilepsy, several mechanisms are involved in the modulation of synaptic transmission. These mechanisms encompass presynaptic regulators responsible for synaptic vesicle formation and release, as well as postsynaptic receptors and neuromodulators that influence the frequency and intensity of epileptic seizures. Moreover, through KEGG pathway enrichment we found the 33 FDEGS were mostly associated with legionellosis, HIF-1 signaling pathway, and autophagy-animal. A case report showed a patient with *legionella longbeachae* pneumonia developed epileptic seizure after using moxifloxacin and provided a possible connection between epileptic and legionellosis (22). Li et al. (23) indicated that the HIF-1α–Notch signaling pathway plays a crucial role in enhancing neurogenesis during acute epilepsy. Furthermore, they observed a reduction in neurogenesis during epileptogenesis when this pathway was blocked. Previous research found electroacupuncture treatment promotes autophagy during epilepsy onset by significantly downregulating the AKT/mTOR signaling pathway, which is consistent with our research (24). Then, we conducted the GSEA, and the result showed that the top 4 pathways were T-cell receptor signaling pathway, cytokine receptor interaction, ribosome, and natural killer cell-mediated cytotoxicity. These results may provide ideas for our future research. GSVA based on known gene sets (25), including KEGG pathways and GO functional terms, evaluated the enrichment level of these gene sets by calculating the overall pattern variation of gene expression in two groups, which helps us understand the relationship between gene sets, diseases, and biological processes.

Furthermore, the PPI network revealed three key FDEGs, namely, HIF1A, TLR4, and CASP8. These hub genes have high diagnostic efficiency in epilepsy. Studies have demonstrated that the activation of hypoxia-inducible factor 1 (HIF-1) can enhance the transcription of multidrug transporters, such as P-glycoprotein (P-gp), in astrocytes. As a result, this process reduces the accumulation of antiepileptic drugs in the brain (26). The former study found that the status epilepticus could induce P-gp and EPO-R expression in cortical pyramidal neurons. *In vitro*, excitotoxic stress can also induce the expression of EPO-R and P-gp simultaneously with both HIF-1α and NFkB nuclear translocation in primary cortical neurons (27). P-gp overexpression in the brain is associated with changes in membrane depolarization in refractory epilepsy (28). Moreover, a previous study found that the expression levels of HIF-1α and P-gp were coordinately increased in the hippocampus and temporal lobes of patients with mesial temporal lobe epilepsy (29, 30). Li et al. (31) showed HIF-1α as a direct target of miRNA-153 and may serve as a potential diagnostic biomarker and therapeutic target for refractory epilepsy. Some studies showed that the TLR4 signaling pathway produces an antiepileptic effect. Yang et al. (32) successfully established an epileptic model and built a pharmacological network, which found that the hippocampus experienced in the epilepsy group contained an upregulation of TLR4, MYD88, and Caspase-3 compared with the control group counterparts. Zhu et al. (33) showed that blocking TLR4/MYD88 signaling attenuated KA-induced neuroinflammation and neuronal damage in the hippocampus.

Recent research demonstrated that after the termination of seizures, the cleavage of (p18) caspase-8 can be detected, along with an increase in the cleavage of the substrate Ile-Glu-Thr-Asp (IETD)-nitroanilide and the appearance of cleaved (p15) Bid. *In vivo* experiments, intraventricular administration of z-iETD-fluoromethylketone significantly reduces the activity of caspase 8, 9, and 3 induced by seizures as well as decreases the cleavage of Bid and caspase-9 and neuronal death. The study showed that intervention targeting caspase-8 and/or the death receptor signaling pathway may protect the brain from injury caused by seizures (34). According to our study, these hub genes were possibly associated with ferroptosis-dependent epilepsy, which still needs more research.

Neuroinflammation is thought to be a contributing factor to epilepsy, and ferroptosis is also reported to be associated with immune disease (35). In our study, we investigated the presence of immune cells and immune infiltration in epilepsy, which revealed significant differences in certain immune cell populations between the epilepsy group and the control group. Compared with the normal group, the CD4-naïve T cells, gamma delta T cells, M1 macrophages, and neutrophils showed low expression in the epilepsy group. It is worth noting that recent research carried out by Xu et al. showed that γδ T lymphocytes producing the pro-inflammatory cytokine IL-17 are concentrated in the epileptogenic zone and positively correlated with the severity of epilepsy. In contrast, the number of infiltrating regulatory T cells (Tregs) in the brain is negatively correlated with disease severity (36). A systematic review showed that the neutrophil-to-lymphocyte ratio (NLR) in epilepsy was higher than in healthy controls. Moreover, elevated NLR value was a good biomarker of inflammation (37). The results were different from our study, possibly because this study is about the acute phase of epilepsy. We also found a significant difference between mast cell-activated cells and 20 other immune cells. Previous research indicated that mast cells activated cells may provide protective activity against seizure mediated by serotonin (38). In addition, Francesco Girolamo et al. proved mast cells influenced by microbes have been shown to promote neuroprotection in neurodegenerative disorders (39). Our study proved the three hub genes were significantly correlated with monocytes (40), neutrophils (41), CD4-naïve T cells, and follicular helper T cells (42). Based on their association, hub genes may profoundly affect epilepsy by regulating immune cell infiltration and modulating inflammation (43, 44).

Previous studies found that ferroptosis impacts immune cells in two fundamentally different ways. On the one hand, ferroptosis affects the number and function of the immune cells themselves. On the other hand, ferroptotic cells can be recognized by immune cells, and then, they trigger a range of inflammatory or specific responses. In our study, we also explored the connection between immune cells and ferroptosis-related genes. An imbalance of macrophage M1/M2 polarization contributes to various diseases or inflammatory conditions (45). Mounting evidence suggests that macrophage polarization and ferroptosis can influence each other at the cell-autonomous level or by communication with other cells in a context-dependent manner. Infiltrating monocytes have also been suggested to promote inflammation after status epilepticus (40). It is possible that alternative monocyte-derived populations, like differentiated macrophages, may affect seizure severity and hippocampal damage in different ways. In our research, M1 macrophage in the epilepsy group were higher than in the normal group, which may be the result of increased TLR4 leading to decreased M1 macrophages and affecting their ability to clear ferroptosis cells, ultimately exacerbating epilepsy (46). Previous several *in vitro* and *in vivo* observations suggest that the activity and function of cytotoxic T cells (CD8) and helper T cells (CD4) are regulated by lipid peroxidation and ferroptosis. Moreover, the study identified that CD4 T cells in the peripheral blood are present in a higher proportion of epilepsy patients, which means CD4 T cells may play an important role in the occurrence and development of epilepsy. Neutrophil granulocytes are the first immune cells recruited to sites of inflammation, and the current evidence suggests that neutrophils participate in sustaining inflammation caused by ferroptotic tissue damage (47). Moreover, a study reveals neutrophils and TNFα as central regulators of neuronal hyperexcitability of diverse etiology in human epilepsy. However, the specific roles of these immune cells in epilepsy and ferroptosis remain unclear. Therefore, further research is urgently needed to elucidate the underlying mechanisms of these immune cells in epilepsy. This research may contribute to identifying new therapeutic or adjunctive treatment strategies for patients with epilepsy.

Despite the remarkable sense, there are also many limitations in our study. First, the data were from the GEO database, including patients with tumors and patients in the restore stage, and our results take the intersection of the different databases. As the original dataset did not provide complete details of other comorbidities or diseases, it is difficult to guarantee that the epilepsy patients included in our analysis were free from other illnesses. Therefore, it poses a challenge to accurately assess and isolate the influence of other diseases on our results. Second, despite integrating multiple epilepsy microarray datasets, the sample size remained limited; increasing the sample size can enhance the statistical power to detect hub genes and immune cells. In addition, although three target genes were identified, it is indeed necessary to conduct wet lab experiments to validate them. By validating the target genes through wet lab experiments, more specific and reliable results can further support our research findings.

## 5. Conclusion

In this study, we successfully identified and validated differentially expressed genes (DEGs) specific to epilepsy that are associated with ferroptosis. This finding enhances our understanding of the molecular mechanisms underlying epilepsy and their relationship to the process of ferroptosis. Additionally, our analysis revealed significant differences in immune infiltration between the epilepsy group and the normal control group, highlighting the potential role of the immune system in epilepsy pathogenesis. Furthermore, our study employed bioinformatics approaches to predict target compounds, drugs, miRNAs, and transcription factors that may be implicated in epilepsy. These predictions offer valuable insights for future research aiming to elucidate the exact pathogenic mechanisms underlying epilepsy and provide potential targets for the development of innovative treatment strategies.

## Data availability statement

The original contributions presented in the study are included in the article/Supplementary material, further inquiries can be directed to the corresponding author.

## Ethics statement

Ethical approval was not required for the study involving humans in accordance with the local legislation and institutional requirements. Written informed consent to participate in this study was not required from the participants or the participants’ legal guardians/next of kin in accordance with the national legislation and the institutional requirements.

## Author contributions

DX: Data curation, Formal analysis, Methodology, Writing – original draft, Writing – review & editing, Software. MC: Writing – review & editing, Investigation, Methodology, Validation. YC: Writing – review & editing, Data curation, Formal analysis. YF: Writing – review & editing, Methodology, Software, Visualization. JW: Investigation, Supervision, Writing – review & editing. XZ: Writing – review & editing, Formal analysis, Supervision. FX: Writing – review & editing, Conceptualization, Project administration.

## Footnotes

^1^ http://www.targetscan.org/vert_71/

^2^ https://dgidb.org/

^3^ https://ctdbase.com
