# Supplementary material for: Identification and verification of ferroptosis-related genes in the pathology of epilepsy: insights from CIBERSORT algorithm analysis
Source: Front Neurol. 2023 Oct 31;14:1275606. doi: 10.3389/fneur.2023.1275606 (PMC10644861; doi:10.3389/fneur.2023.1275606)
Supplement: Supplementary file 1 [file Table_1.docx]

**TableS1 Ferroptosis related genes**

| **GeneCards** |
| --- |
| **AIFM2** |
| **GPX4** |
| **TP53** |
| **ACSL4** |
| **HMOX1** |
| **NFE2L2** |
| **BECN1** |
| **SLC7A11** |
| **TFRC** |
| **MAP1LC3B** |
| **VDAC2** |
| **VDAC3** |
| **ALOX15** |
| **LINC00336** |
| **PRKAA1** |
| **ATG5** |
| **ATG7** |
| **NCOA4** |
| **LAMP2** |
| **SLC3A2** |
| **PARK7** |
| **PCBP1** |
| **MDM2** |
| **BAP1** |
| **HMGB1** |
| **VDAC1** |
| **TF** |
| **NF2** |
| **YAP1** |
| **CBS** |
| **PRDX6** |
| **IDH1** |
| **PTEN** |
| **TNFAIP3** |
| **SREBF1** |
| **WWTR1** |
| **PEBP1** |
| **AHCY** |
| **STYK1** |
| **SESN2** |
| **PVT1** |
| **MIR214** |
| **IDH2** |
| **JUN** |
| **HNRNPA1** |
| **MDM4** |
| **ARNTL** |
| **CISD1** |
| **PROM2** |
| **LONP1** |
| **HDDC3** |
| **POR** |
| **AKR1C2** |
| **AKR1C3** |
| **CCL5** |
| **AKR1C1** |
| **ABCB10** |
| **CYB5R1** |
| **MIR326** |
| **ABCB6** |
| **NEDD4** |
| **PLIN2** |
| **PANX1** |
| **GABPB1** |
| **GABPB1-AS1** |
| **CA9** |
| **DNAJB6** |
| **SAT1** |
| **ELAVL1** |
| **TNF** |
| **FBXW7** |
| **ATG16L1** |
| **ZFP36** |
| **CD44** |
| **ATF4** |
| **ITGA6** |
| **PRKAA2** |
| **FANCD2** |
| **ALOX12** |
| **MAPK1** |
| **MYC** |
| **EGLN1** |
| **MAP3K5** |
| **GOT1** |
| **FH** |
| **HELLS** |
| **SOCS1** |
| **OTUB1** |
| **CARS1** |
| **MIR9-1** |
| **MIR137** |
| **HSPB1** |
| **HSPA5** |
| **NFS1** |
| **RB1** |
| **G3BP1** |
| **LINC00472** |
| **MT1G** |
| **EPAS1** |
| **HIF1A** |
| **G0S2** |
| **HILPDA** |
| **PLA2G6** |
| **PRC1** |
| **TGFBR1** |
| **UCHL1** |
| **GSTP1** |
| **VIM** |
| **FASN** |
| **ANXA1** |
| **MYH9** |
| **YWHAE** |
| **YWHAG** |
| **ENO2** |
| **ALDOA** |
| **HSPA9** |
| **GNB3** |
| **PGK1** |
| **YWHAB** |
| **YWHAZ** |
| **TUBA1A** |
| **RPL5** |
| **ACVR1B** |
| **DLST** |
| **CFL1** |
| **HSP90B1** |
| **HADHA** |
| **LDHB** |
| **GPI** |
| **PRDX2** |
| **MYH10** |
| **UBA1** |
| **XRCC6** |
| **DPYSL2** |
| **CKB** |
| **RPL10** |
| **RPA2** |
| **RUVBL1** |
| **PDIA3** |
| **TUBB4A** |
| **EIF4A1** |
| **SMC3** |
| **VAPB** |
| **EWSR1** |
| **CCT5** |
| **HP** |
| **RPL15** |
| **RPSA** |
| **VAPA** |
| **XRCC5** |
| **CBX3** |
| **FSCN1** |
| **TRIM28** |
| **BANF1** |
| **ACTC1** |
| **DUT** |
| **CNBP** |
| **KHDRBS1** |
| **LRPPRC** |
| **HNRNPD** |
| **RPL26** |
| **RPS7** |
| **RPL13A** |
| **RPL7** |
| **RPS13** |
| **PDIA4** |
| **PTBP1** |
| **CCT3** |
| **MAP4** |
| **HMGN1** |
| **PDIA6** |
| **RPS3A** |
| **RPLP0** |
| **RPLP2** |
| **PFN2** |
| **CCT4** |
| **DHX9** |
| **HNRNPM** |
| **ITIH3** |
| **KSR2** |
| **MYL6B** |
| **RPL30** |
| **RPL34** |
| **SNRPA** |
| **SNRPD2** |
| **PLXNA4** |
| **SSBP1** |
| **CCT6A** |
| **CCT8** |
| **HNRNPL** |
| **RPS4X** |
| **SNRPD1** |
| **SLC25A31** |
| **YBX1** |
| **WTAP** |
| **HNRNPA3** |
| **NACA** |
| **NOLC1** |
| **NAP1L1** |
| **NGB** |
| **RUFY1** |
| **SNRPC** |
| **SNRPF** |
| **USP44** |
| **ALYREF** |
| **ERH** |
| **CNTNAP4** |
| **FAM120A** |
| **MUC16** |
| **RPL38** |
| **RACK1** |
| **TBC1D5** |
| **ATP5F1A** |
| **RPL36A** |
| **TEX264** |
| **PCBP3** |
| **YTHDF3** |
| **WBP11** |
| **ACTBL2** |
| **RUFY3** |
| **ATP5F1B** |
| **SNU13** |
| **IGKC** |
| **RTL1** |
| **KBTBD3** |
| **KIAA1614** |
| **SRSF8** |
| **H2AC1** |
| **ABRAXAS2** |
| **IGHG4** |
| **H2AC21** |
| **TUBA4B** |
| **IGKV1-5** |
| **IGLV3-19** |
| **FTH1** |
| **CP** |
| **FTL** |
| **SLC11A2** |
| **GSS** |
| **CYBB** |
| **PRNP** |
| **SLC40A1** |
| **ACSL1** |
| **GCLC** |
| **STEAP3** |
| **MAP1LC3A** |
| **SLC39A14** |
| **SLC39A8** |
| **ACSL5** |
| **GCLM** |
| **ACSL3** |
| **PCBP2** |
| **ACSL6** |
| **SAT2** |
| **FTMT** |
| **LPCAT3** |
| **MAP1LC3C** |
| **MAP1LC3B2** |
| **SIAH2** |
| **TIGAR** |
| **AURKA** |
| **CASP8** |
| **RIPK1** |
| **CDKN2A** |
| **MIR4715** |
| **CFTR** |
| **MIF** |
| **MUC1** |
| **ALOX15B** |
| **DECR1** |
| **ANO6** |
| **RNF113A** |
| **GUCY1A1** |
| **MIR7-1** |

| **FerrDb** |
| --- |
| **RPL8** |
| **IREB2** |
| **ATP5MC3** |
| **CS** |
| **EMC2** |
| **ACSF2** |
| **NOX1** |
| **CYBB** |
| **NOX3** |
| **NOX4** |
| **NOX5** |
| **DUOX1** |
| **DUOX2** |
| **G6PD** |
| **PGD** |
| **VDAC2** |
| **PIK3CA** |
| **FLT3** |
| **SCP2** |
| **TP53** |
| **ACSL4** |
| **LPCAT3** |
| **NRAS** |
| **KRAS** |
| **HRAS** |
| **TF** |
| **TFRC** |
| **TFR2** |
| **SLC38A1** |
| **SLC1A5** |
| **GLS2** |
| **GOT1** |
| **CARS1** |
| **ALOX5** |
| **KEAP1** |
| **HMOX1** |
| **ATG5** |
| **ATG7** |
| **NCOA4** |
| **ALOX12** |
| **ALOX12B** |
| **ALOX15** |
| **ALOX15B** |
| **ALOXE3** |
| **PHKG2** |
| **ACO1** |
| **G6PDX** |
| **ULK1** |
| **ATG3** |
| **ATG4D** |
| **BECN1** |
| **MAP1LC3A** |
| **GABARAPL2** |
| **GABARAPL1** |
| **ATG16L1** |
| **WIPI1** |
| **WIPI2** |
| **SNX4** |
| **ATG13** |
| **ULK2** |
| **SAT1** |
| **EGFR** |
| **MAPK3** |
| **MAPK1** |
| **BID** |
| **ZEB1** |
| **DPP4** |
| **CDKN2A** |
| **PEBP1** |
| **SOCS1** |
| **CDO1** |
| **MYB** |
| **MAPK8** |
| **MAPK9** |
| **CHAC1** |
| **MAPK14** |
| **LINC00472** |
| **PRKAA2** |
| **PRKAA1** |
| **ELAVL1** |
| **BAP1** |
| **ABCC1** |
| **MIR6852** |
| **ACVR1B** |
| **TGFBR1** |
| **EPAS1** |
| **HILPDA** |
| **HIF1A** |
| **IFNG** |
| **ANO6** |
| **LPIN1** |
| **HMGB1** |
| **TNFAIP3** |
| **TLR4** |
| **ATF3** |
| **ATM** |
| **YY1AP1** |
| **EGLN2** |
| **MIOX** |
| **TAZ** |
| **MTDH** |
| **IDH1** |
| **SIRT1** |
| **FBXW7** |
| **PANX1** |
| **DNAJB6** |
| **BACH1** |
| **LONP1** |
| **PTGS2** |
| **DUSP1** |
| **NOS2** |
| **NCF2** |
| **MT3** |
| **UBC** |
| **ALB** |
| **TXNRD1** |
| **SRXN1** |
| **GPX2** |
| **BNIP3** |
| **OXSR1** |
| **SELENOS** |
| **ANGPTL7** |
| **SLC7A11** |
| **DDIT4** |
| **LOC284561** |
| **ASNS** |
| **TSC22D3** |
| **DDIT3** |
| **JDP2** |
| **SESN2** |
| **SLC1A4** |
| **PCK2** |
| **TXNIP** |
| **VLDLR** |
| **GPT2** |
| **PSAT1** |
| **LURAP1L** |
| **SLC7A5** |
| **HERPUD1** |
| **XBP1** |
| **SLC3A2** |
| **CBS** |
| **ATF4** |
| **ZNF419** |
| **KLHL24** |
| **TRIB3** |
| **ZFP69B** |
| **ATP6V1G2** |
| **VEGFA** |
| **GDF15** |
| **TUBE1** |
| **ARRDC3** |
| **CEBPG** |
| **SNORA16A** |
| **RGS4** |
| **BLOC1S5-TXNDC5** |
| **LOC390705** |
| **EIF2S1** |
| **KIM-1** |
| **IL6** |
| **CXCL2** |
| **RELA** |
| **HSD17B11** |
| **AGPAT3** |
| **SETD1B** |
| **FTL** |
| **MAFG** |
| **IL33** |
| **FTH1** |
| **SLC40A1** |
| **GPX4** |
| **HAMP** |
| **HSPB1** |
| **NFE2L2** |
| **STEAP3** |
| **DRD5** |
| **DRD4** |
| **MAP3K5** |
| **SLC2A1** |
| **SLC2A3** |
| **SLC2A6** |
| **SLC2A8** |
| **SLC2A12** |
| **GLUT13** |
| **SLC2A14** |
| **EIF2AK4** |
| **TFAP2C** |
| **SP1** |
| **HBA1** |
| **NNMT** |
| **PLIN4** |
| **HIC1** |
| **STMN1** |
| **RRM2** |
| **CAPG** |
| **HNF4A** |
| **NGB** |
| **YWHAE** |
| **GABPB1** |
| **AURKA** |
| **MIR4715** |
| **RIPK1** |
| **PRDX1** |
| **MIR30B** |
| **AKR1C1** |
| **AKR1C2** |
| **AKR1C3** |
| **RB1** |
| **HSF1** |
| **GCLC** |
| **SQSTM1** |
| **NQO1** |
| **MUC1** |
| **MT1G** |
| **CISD1** |
| **FANCD2** |
| **FTMT** |
| **HSPA5** |
| **HELLS** |
| **SCD** |
| **FADS2** |
| **SRC** |
| **STAT3** |
| **PML** |
| **MTOR** |
| **NFS1** |
| **TP63** |
| **CDKN1A** |
| **MIR137** |
| **ENPP2** |
| **FH** |
| **CISD2** |
| **MIR9-1** |
| **MIR9-2** |
| **MIR9-3** |
| **ISCU** |
| **ACSL3** |
| **OTUB1** |
| **CD44** |
| **LINC00336** |
| **BRD4** |
| **PRDX6** |
| **MIR17** |
| **NF2** |
| **ARNTL** |
| **JUN** |
| **CA9** |
| **TMBIM4** |
| **PLIN2** |
| **MIR212** |
| **Fer1HCH** |
| **AIFM2** |
| **LAMP2** |
| **ZFP36** |
| **PROM2** |
| **CHMP5** |
| **CHMP6** |
| **CAV1** |
| **GCH1** |
